# Association Between Antidiarrheal Drug Prescription and Return Visits Among Adult Patients With Acute Diarrhea

**DOI:** 10.7759/cureus.18807

**Published:** 2021-10-15

**Authors:** Yasuhiro Osugi, Kenichiro Ishiguro, Daiki Kobayashi

**Affiliations:** 1 General Medicine, Toyota Regional Medical Center, Toyota, JPN; 2 Internal Medicine, Ishiguro Home-Visiting Clinic, Toyota, JPN; 3 Internal Medicine, Seiroka Kokusai Byouin, Tsukiji, JPN

**Keywords:** japan, acute diarrhea, admission, return visit, antidiarrheal drug

## Abstract

Whether antidiarrheal medications have benefits or demerits when administered to adult patients with diarrhea remains controversial. We aimed to evaluate the association between antidiarrheal drug prescription and clinical outcomes in adult patients with acute diarrhea.

This retrospective cohort study was conducted by collecting secondary data of patients' health records at St. Luke’s International Hospital from April 1, 2004, to March 31, 2016. We included all participants aged 20-59 years who visited the division of general internal medicine or the emergency room in the hospital due to acute diarrheal symptoms. We excluded those who had chronic diarrhea or were immunocompromised (e.g., those with cancer or immunosuppressant usage). Our primary outcome was return visits within two weeks; the secondary outcome was admission to the hospital due to acute diarrhea within two weeks from the first visit. We compared the outcomes between patients with and without antidiarrheal drug prescriptions.

During the study period, a total of 10,246 patients were included, of which 204 (2.0%) were prescribed antidiarrheal drugs. The mean age of the patients was 35.0 (standard deviation: 10.7) years, and 4,130 (40.3%) were men. Patients who were prescribed antidiarrheal drugs were more likely to be prescribed antibiotics (p<0.01). The adjusted odds ratios for return visits among patients with and without antidiarrheal drug prescription were 1.24-1.59, which were not significant.

We demonstrated that antidiarrheal drug prescription was not associated with return visits or hospital admission among adult patients with acute diarrhea. This finding suggests that antidiarrheal medications have more benefits than risks in adult patients with acute diarrhea.

## Introduction

Acute diarrhea is one of the most common diseases in both low- and high-income countries [1]. Although patients may or may not require antibiotic treatment for bacterial diarrhea, symptomatic treatments are common for all patients with acute diarrhea [2]. As a symptomatic treatment, antidiarrheal medications are sometimes prescribed for patients with acute watery diarrhea or noninfectious diarrhea based on moderate-quality evidence [3]. However, there are arguments about the possible side effects of antidiarrheal drugs for adults patients. Especially for pediatric patients, the antidiarrheal medications may worsen the condition, causing hemolytic uremic syndrome (HUS) [4]. Furthermore, it remains controversial whether antidiarrheal medications have benefits or demerits when administered to adult patients with diarrhea [5]. Therefore, we aimed to evaluate the association between antidiarrheal drug prescription and clinical outcomes, such as return visits or admission, in adult patients with acute diarrhea.

## Technical report

This retrospective cohort study was conducted at St. Luke’s International Hospital from April 1, 2004, to March 31, 2016. We included all participants aged 20-59 years who visited the division of general internal medicine or the emergency room in the hospital due to acute diarrheal symptoms. We excluded those who had chronic diarrhea or were immunocompromised (e.g., those with cancer or immunosuppressant usage). Our primary outcome was return visits within two weeks; the secondary outcome was admission to the hospital due to acute diarrhea within two weeks from the first visit based on the previous study [6]. We compared the outcomes between patients with and without antidiarrheal drug prescriptions. Antidiarrheal drugs were defined based on the Anatomical Therapeutic Chemical classification (diphenoxylate, opiates, loperamide, difenoxin, loperamide oxide, albumin tannate, Ceratonia, and racecadotril) [7]. After providing descriptive statistics based on the prescription status of antidiarrheal drugs, we performed logistic regression analysis adjusting for the potential confounders. St. Luke’s International Hospital Ethical Committee approved this study (17-R177).

During the study period, a total of 10,246 patients were included, of which 204 (2.0%) were prescribed antidiarrheal drugs. The mean age of the patients was 35.0 (standard deviation: 10.7) years, and 4,130 (40.3%) were men (Table 1).

**Table 1:** Characteristics and clinical outcomes of patients with and without antidiarrheal drug prescription SD: standard deviation; bpm: beats per minute

	Antidiarrheal drug prescription (n=206)		No antidiarrheal drug prescription (n=10,040)		p-value	Total (n=10,246)	
Demographics							
Age, year, mean (SD)	33.8	9.9	35.0	10.7	0.12	35.0	10.7
Male, n (%)	91	44.2	4,039	40.2	0.25	4,130	40.3
Vital signs							
Body temperature, ℃, mean (SD)	36.8	0.8	37.0	1.4	0.48	37.0	1.4
Heart rate, bpm, mean (SD)	84.5	16.7	86.3	17.1	0.50	86.3	17.1
Systolic blood pressure, mmHg, mean (SD)	115.1	22.2	119.1	24.7	0.28	119.1	24.7
Diastolic blood pressure, mmHg, mean (SD)	67.0	17.9	72.3	19.8	0.07	72.2	19.8
Antibiotic prescription	42	20.4	908	9.0	<0.01	950	9.3
Outcomes							
Return visits within 2 weeks	23	11.2	935	9.3	0.37	958	9.4
Admission within 2 weeks	0	0.0	231	2.3	0.03	231	2.3

Patients who were prescribed antidiarrheal drugs were more likely to be prescribed antibiotics (p<0.01). Table 2 shows the results of multivariable logistic regression analysis for return visits within two weeks with different covariates.

**Table 2:** Results of multivariable logistic regression analysis for return visits within 2 weeks *Model 1 was adjusted for age and sex; model 2 was adjusted for vital signs in addition to model 1 variables; model 3 was adjusted for prescription of antibiotics in addition to model 2 variables.

	Adjusted odds ratio (95% confidence interval)		
	Model 1^*^	Model 2	Model 3
Antidiarrheal drug prescription	1.24 (0.80–1.93)	1.57 (0.65–3.79)	1.59 (0.66–3.84)
Age	1.01 (1.01–1.02)	1.01 (0.99–1.02)	1.01 (0.99–1.02)
Sex (male)	1.07 (0.94–1.23)	1.01 (0.80–1.27)	1.00 (0.79–1.26)
Body temperature	―	1.06 (0.99–1.13)	1.06 (0.99–1.13)
Heart rate	―	0.99 (0.99–1.00)	0.99 (0.99–1.00)
Systolic blood pressure	―	1.00 (1.00–1.01)	1.00 (1.00–1.01)
Antibiotic prescription	―	―	2.20 (1.43–3.39)

The adjusted odds ratios for return visits among patients with and without antidiarrheal drug prescription were 1.24-1.59, which was not significant.

## Discussion

We demonstrated that antidiarrheal drug prescription was not associated with return visits or hospital admission among adult patients with acute diarrhea. This finding suggests that antidiarrheal medications have more benefits than risks in adult patients with acute diarrhea. Moreover, a very limited number of HUS cases has been reported among adult patients with diarrhea who were prescribed antidiarrheal medications [8]. Patients who were prescribed antibiotics had high odds of return visits, possibly because of severe symptoms. As prescription of antibiotics completely depended on physicians’ decisions, patients with severe symptoms, such as a large volume of diarrhea or severe abdominal pain, may be more likely to be prescribed antibiotics [9]. Therefore, patients with antibiotic usage may have severe symptoms and may be more likely to return or be admitted to the hospital [10]. One of the limitations of our study was that our data did not include some clinical data, such as the severity of abdominal pain or the volume of diarrhea, which may have affected the clinical outcomes. Also, the very low number of antidiarrheal drug prescriptions and the very big difference between the two comparison groups weaken the results. In addition, the single medical center study was one of the major limitations.

## Conclusions

In conclusion, antidiarrheal medications may not be disadvantageous in adult patients with acute diarrhea. Physicians may be able to prescribe antidiarrheal medications especially for adult diarrheal patients with severe symptoms.
